# Cell-penetrating artificial mitochondria-targeting peptide-conjugated metallothionein 1A alleviates mitochondrial damage in Parkinson’s disease models

**DOI:** 10.1038/s12276-018-0124-z

**Published:** 2018-08-17

**Authors:** Young Cheol Kang, Minuk Son, Sora Kang, Suyeol Im, Ying Piao, Kwang Suk Lim, Min-Young Song, Kang-Sik Park, Yong-Hee Kim, Youngmi Kim Pak

**Affiliations:** 1 Department of Neuroscience, Graduate School, Kyung Hee University, Seoul, 02447 Korea; 2 Department of Physiology, College of Medicine, Kyung Hee University, Seoul, 02447 Korea; 3 Department of Bioengineering, Institute for Bioengineering and Biopharmaceutical Research, Hanyang University, Seoul, 133-791 Korea; 4 Biomedical Omics Group, Korea Basic Science Institute, Cheongju-si, Chungbuk South Korea; 5 Present Address: Department of Emergency, Yanbian University Hospital, Yanji City, Jilin Province China

## Abstract

An excess of reactive oxygen species (ROS) relative to the antioxidant capacity causes oxidative stress, which plays a role in the development of Parkinson’s disease (PD). Because mitochondria are both sites of ROS generation and targets of ROS damage, the delivery of antioxidants to mitochondria might prevent or alleviate PD. To transduce the antioxidant protein human metallothionein 1A (hMT1A) into mitochondria, we computationally designed a cell-penetrating artificial mitochondria-targeting peptide (CAMP). The recombinant CAMP-conjugated hMT1A fusion protein (CAMP-hMT1A) successfully localized to the mitochondria. Treating a cell culture model of PD with CAMP-hMT1A restored tyrosine hydroxylase expression and mitochondrial activity and reduced ROS production. Furthermore, injection of CAMP-hMT1A into the brain of a mouse model of PD rescued movement impairment and dopaminergic neuronal degeneration. CAMP-hMT1A delivery into mitochondria might be therapeutic against PD by alleviating mitochondrial damage, and we predict that CAMP could be used to deliver other cargo proteins to the mitochondria.

## Parkinson’s disease: restoring mitochondrial function in disease models

A peptide targeting mitochondria can help deliver an antioxidant protein to mitigate the effects of Parkinson’s disease in cellular and mouse models. Youngmi Pak from Kyung Hee University, Seoul, South Korea, and co-workers engineered bacteria to express the human version of an antioxidant protein called metallothionein 1A fused to a short peptide sequence so that it localizes to mitochondria, the cellular power plants. Once inside the mitochondria, the peptide is removed, leaving the mature antioxidant protein to mop up damaging free radicals, a common problem seen in the cells of patients with Parkinson’s disease, and restore mitochondria to a healthier state. The protein improved mitochondrial function in both a human cell line and in the neurons of mice with a Parkinson’s-like disease, suggesting it might also help patients with this devastating neurological condition.

## Introduction

Parkinson’s disease (PD) is a progressive neurodegenerative disease that is characterized by the selective degeneration of dopaminergic neurons in the substantia nigra (SN) pars compacta (SNpc) and their axon terminals that project to the dorsal striatum (ST)^1^. A concomitant reduction of the striatal concentration of dopamine (DA) results in difficulties in performing both voluntary and involuntary movements, such as bradykinesia, tremors, cogwheel rigidity, and postural instability^2^. Although the causes of PD are still unclear, the evidence strongly suggests that mitochondrial dysfunction and oxidative stress are involved in its pathogenesis^3,4^. The PD-associated PARK genes associated with familial PD, such as *PINK1*, *Parkin*, *DJ-1*, *PARIS*, and *LRRK2*, are implicated in various aspects of mitochondrial biology, including bioenergetic capacity, reactive oxygen species (ROS) accumulation, quality control, morphologic changes, and mitochondrial protein transport^5,6^. Mutations in PARK genes and the mitochondrial DNA (mtDNA) polymerase γ are found in the SN region of PD patients^7^. Notably, the activity of complex 1 (NADH-ubiquinone oxidoreductase) in mitochondrial oxidative phosphorylation (OXPHOS) is decreased in post-mortem SN^8^. Moreover, OXPHOS inhibitors, such as rotenone, paraquot, 1-methyl-4-phenyl-1,2,3,6-tetrahydropyridine (MPTP), and 1-methyl-4-phenylpyridinium (MPP^+^), induce the selective loss of dopaminergic neurons and are used to generate animal or cellular models of PD to evaluate candidate therapies^9^.

Mitochondria serve a critical role in generating the ATP required for cell viability via OXPHOS. Mitochondrial OXPHOS also continuously generates ROS as a byproduct of the consumption of molecular oxygen reacting with electrons leaked from the electron transport chain. Under normal conditions, the level of ROS is finely controlled by enzymatic and non-enzymatic mechanisms, responding to cellular demands. On the other hand, the inhibition of OXPHOS produces an excess amount of ROS that leads to damage in mtDNA, components of the respiratory chain, and other mitochondrial factors if the ROS are not scavenged by endogenous antioxidants, thereby triggering a vicious cycle of mitochondrial impairment and oxidative stress, resulting in many diseases^4^. Therefore, controlling the cellular ROS balance by introducing selective antioxidants into mitochondria may be a promising therapeutic path for treating diseases in which mitochondrial damage is involved.

Protein transduction domains (PTDs), also known as cell-penetrating peptides, allow the in vivo delivery of bioactive cargo such as proteins, nucleic acids, and nanoparticles across the membrane and blood-brain barrier^10^. The human immunodeficiency virus (HIV-1) *trans*-activator of transcription (TAT) peptide is a well-known PTD that is an arginine- and lysine-rich 11-amino-acid peptide (YGRKKRRQRRR)^11,12^. TAT-fusion proteins are rapidly and efficiently introduced into cultured cells or intact tissues while retaining their biological activities^13^. Subsequent studies have shown that the TAT-mediated transduction into targeted cells occurs in a concentration-dependent and receptor-, transporter-, and endocytic-independent manner^14^. Moreover, when a known mitochondria-targeting sequence (MTS) is present in the cargo protein, the fusion proteins are found in the mitochondria^15,16^.

Most mitochondrial proteins are synthesized in the cytosol as precursors with a 10- to 70-amino-acid-long MTS at the N terminus, which is recognized by translocation machinery at mitochondrial membrane contact sites and allows the precursor to be imported into the mitochondria^17^. MTSs are able to form strong basic amphipathic alpha-helices that are essential for efficient mitochondrial transport, containing 3–5 nonconsecutive basic Arg/Lys (R/K) residues and often several Ser/Thr (S/T) residues, without any acidic Asp/Glu (D/E) residues. Upon entering the mitochondria, the MTS is cleaved off by matrix-processing proteases at a well-conserved Rxx_(S/A) site^18,19^. Therefore, PTD and MTS both employ basic and amphiphilic helical structures to enhance the cellular uptake and mitochondrial import of cargo proteins in mammalian cells.

Metallothionein (MT) is a candidate antioxidant protein for easy transfer into the mitochondria because of its small size (6–7 kDa, 61–68 amino acids)^20,21^. Mammalian MT contains 20 cysteine residues and lacks aromatic amino acids. Because each MT protein molecule binds 7–12 heavy metal atoms through its cysteine residues, it has been proposed to be useful for removing heavy metals such as copper (Cu^2+^), zinc (Zn^2+^), and cadmium (Cd^2+^). MT can also scavenge a wide range of ROS (e.g., superoxide, hydrogen peroxide, hydroxyl radical, and nitric oxide) more efficiently than other antioxidants^22^. In many types of cells, MT expression is induced by high concentrations of heavy metals or cell stress conditions. MTs are known to localize to the cytoplasm, lysosomes, and mitochondrial intermembrane space. Alternative splicing generates four groups of human MTs (hMTs): hMT1 and hMT2 are ubiquitously expressed in most tissues, including the central nervous system (CNS), whereas hMT3 and hMT4 are expressed primarily in the CNS and epithelium^23^. Posttranslational acetylation and/or variations in metal composition result in a heterogeneity of hMT1 isoforms. The protein level of hMT1A, a major form of hMT1, is increased in pathologic brain specimens and models of neurodegenerative diseases. The overexpression of MT1 blocks neuro-inflammation, neuronal apoptosis, and ROS accumulation^21^, and promotes cell cycle progression, mitosis, and cell survival^24^.

Previously, we prepared a recombinant mouse MT fusion protein (TAT-sMTS-MT), which was fused with TAT and a short MTS peptide (sMTS) derived from the MTS of mitochondria malate dehydrogenase (mMDH). TAT-sMTS-MT was taken up into the cytoplasm of H9c2 cells and showed bioactivity^25^. However, the sMTS neither localized the fusion protein into mitochondria nor significantly prolonged the intracellular retention time of the fusion protein, although it enhanced the intracellular transduction efficiency of the cargo protein.

In the present study, we designed a novel cell-penetrating artificial mitochondria-targeting peptide (CAMP) to deliver proteins to mitochondria by ligating TAT with an artificial MTS. When hMT1A was conjugated to CAMP, this recombinant fusion protein (CAMP-hMT1A) localized to the mitochondria where it was able to eliminate ROS and restore mitochondrial activity in a cell culture PD model. This is the first study to explore the possibility of direct delivery of mitochondria-targeted hMT1A in the context of protein therapy to treat patients with mitochondrial deficiencies.

## Materials and methods

### In silico analyses of artificial MTS peptides

The mitochondria-targeting probabilities of artificial MTS peptides and CAMP-hMT1A fusion proteins were calculated in silico using the MitoProtII prediction program (http://ihg.gsf.de/ihg/mitoprot.html)^26^. The secondary structure of the CAMP peptide was predicted using the Crystallographic Object-Oriented Toolkit (COOT) software^27^.

### Cell culture

SK-Hep1 human hepatocellular carcinoma cells (American Type Cultute Collection (ATCC)# HTB-52) were cultured in high-glucose (4.5 g/l) Dulbecco’s modified Eagle’s medium (DMEM) supplemented with 10% fetal bovine serum (FBS) and antibiotics (100 μg/ml penicillin/streptomycin mix) in a humidified atmosphere at 37 °C with 5% CO_2_. SH-SY5Y human neuroblastoma cells (ATCC# CRL-2266) were cultured in DMEM/F12 supplemented with 10% FBS and antibiotics at 37 °C/5% CO_2_.

### Preparation and transfection of CAMP-hMT1A fusion protein expression plasmids

The synthetic *Hin*dIII-CAMP-hMT1A-6xHis-*Bam*HI gene (Bioneer, Daejeon, Korea) was cloned into the pcDNA3.1 vector (Promega, Madison, WI, USA) for mammalian expression (pCAMP-hMT1A). The sequence of the synthetic gene was *AAG CTT* ATG GGC TAT GGC AGG AAG AAG CGG AGA CAG CGA CGA CGA TTG TTG CGC GCT GCC CTG CGC AAG GCT GCC CTG ATG GAC CCC AAC TGC TCC TGC GCC ACT GGT GGC TCC TGC ACC TGC ACT GGC TCC TGC AAA TGC AAA GAG TGC AAA TGC ACC TCC TGC AAG AAG AGC TGC TGC TCC TGC TGC CCC ATG AGC TGT GCC AAG TGT GCC CAG GGC TGC ATC TGC AAA GGG GCA TCA GAG AAG TGC AGC TGC TGT GCC CAT CAT CAT CAT CAT CAT TAG *GGA TCC*. The PCR fragment of *Nhe*I-CAMP-*Hind*III was cloned into the pcDNA3.1-GFP-N3 vector (Clontech, Mountain View, CA, USA) to prepare the pCAMP-EGFP plasmid. The pCAMP-hMT1A and pCAMP-EGFP plasmids were separately transfected into 70% confluent SK-Hep1 cells in six-well plates using Superfect^®^ transfection reagent (QIAGEN, Valencia, CA, USA). pcDNA3.1-transfected cells were used as a control. SK-Hep1 cells expressing DsRed2-mito were prepared by stable transfection of pDsRed2-mito (Clontech) using the same method^28^. Stably transfected cells were established by selection using G418 (1000 μg/ml) for 2 weeks.

### Preparation of recombinant CAMP-hMT1A protein

The synthetic *Nco*I-CAMP-*Hin*dIII-hMT1A-*Xho*I gene (Bioneer) was cloned into the pET28a(+) vector (Clontech) to prepare the pET28a-CAMP-MT1A bacterial expression plasmid. The sequence of the synthetic gene was *CC ATG G*GC TAT GGC AGG AAG AAG CGG AGA CAG CGA CGA CGA TTG TTG CGC GCT GCC CTG CGC AAG GCT GCC CTG GGA *AAG CTT* ATG GAC CCC AAC TGC TCC TGC GCC ACT GGT GGC TCC TGC ACC TGC ACT GGC TCC TGC AAA TGC AAA GAG TGC AAA TGC ACC TCC TGC AAG AAG AGC TGC TGC TCC TGC TGC CCC ATG AGC TGT GCC AAG TGT GCC CAG GGC TGC ATC TGC AAA GGG GCA TCA GAG AAG TGC AGC TGC TGT GCC *CTC GAG*. The *Hin*dIII-EGFP-*Xho*I and *Nco*I-TAT-*Hin*dIII PCR fragments were cloned into pET28a(+) to prepare the pET28a-TAT-hMT1A and pET28a-CAMP-hMT1A plasmids, respectively.

The pET28a-CAMP-hMT1A plasmid was transformed into *Escherichia coli* strain BL21(DE3)pLysS (Novagen, Madison, WI, USA). The cells were then cultured in LB medium containing 50 μg/ml ampicillin at 37 °C for 4 h, and CAMP-hMT1A protein expression was induced by adding 1 mM isopropyl-β-d-thio-galactoside and incubating overnight at 26 °C. To increase the stability of MT1A, 1 mM ZnSO_4_ (Sigma-Aldrich, St. Louis, MO, USA) was added during the induction. Cell pellets were collected by centrifugation and suspended in lysis buffer (300 mM KCl, 49 mM KH_2_PO_4_, and 4.9 mM imidazole) in the presence of 100 mM phenylmethylsulfonyl fluoride (PMSF), followed by sonication for eight cycles of 30 s each^25^. The supernatant was filtered through a 0.45-μm filter and purified by fast protein liquid chromatography using a Ni-NTA resin immobilized metal affinity chromatography column (Bio-Rad, Hercules, CA, USA). Salt was eliminated from the purified protein by dialysis against pH 7.4 phosphate-buffered saline (PBS) containing 20% glycerol and 1 mM PMSF using a 3500 kDa cutoff membrane (Spectrum Laboratories, Rancho Dominguez, CA, USA). A protease inhibitor cocktail (Roche, Basel, Switzerland) was added to the protein preparations before storage at 4 °C. The fusion proteins were subjected to electrophoresis on 12% SDS-polyacrylamide gel electrophoresis (SDS-PAGE) gels and then stained with Coomassie brilliant blue (Bio-Rad) for 60 min for detection.

### Mass spectrum analysis

Mass spectrometry analysis of CAMP-hMT1A was performed as described previously^29^. The purified CAMP-hMT1A protein gel band was reduced with 5 mg/ml dithiothreitol in 50 mM ammonium bicarbonate (ABC) at 60 °C for 1 h and alkylated by 10 mg/ml iodoacetamide in 50 mM ABC at room temperature for 1 h. The reduced and alkylated protein sample was incubated at 37 °C for 18 h in 50 mM ABC containing 100 μg/ml trypsin. The supernatant peptide mixtures were extracted with 50% ABC in 5% formic acid (FA) for 4 h and dried in a speed vacuum concentrator. The tryptic-dried samples were analyzed using the Agilent HPLC-Chip/TOF MS system with the Agilent 1260 nano-LC system, HPLC Chip-cube MS interface, and 6530 QTOF mass spectrometer (Agilent Technologies, Santa Clara, CA, USA). The dried peptide samples were resuspended in 2% acetonitrile (ACN)/0.1% FA and concentrated on a large-capacity HPLC-Chip (Agilent Technologies). The HPLC-Chip was incorporated into an enrichment column (9 mm, 75 μm inner diameter (I.D.), 160 nl) and a reverse-phase column (15 cm, 75 μm I.D., packed with Zorbax 300SB-C18 5-μm resin). The peptide separation was performed using a 110-min gradient of 3-45% buffer B (buffer A: 0.1% FA, buffer B: 90% ACN/0.1% FA) at a flow rate of 300 nl/min. The mass spectrometry (MS) and tandem mass spectrometry (MS/MS) data were acquired in the positive ion mode, and the data were stored in centroid mode. The chip spray voltage was set at 1900 V and maintained under chip conditions. The drying gas temperature was set to 325 °C with a flow rate of 3.5 l/min. A medium (4 *m*/*z*) isolation window was used for precursor isolation. Collision energy with a slope of 3.7 V/100 Da and an offset of 2.5 V was used for fragmentation. The MS data were acquired over a mass range of 300–3000 *m*/*z*, and the MS/MS data were acquired over a mass range of 50–2500 *m*/*z*. Reference mass correction was performed using a reference mass of 922.0098. The precursors were set in an exclusion list for 0.5 min after two MS/MS spectra. The MS/MS spectra were extracted using the MassHunter Qualitative Analysis B.05.00 software (Agilent Technologies) with default parameters, and the spectra were interpreted with Mascot v2.3 (Matrix Science, London, UK) by searching against the Uniprot database (18/11/2011). Individual ion scores >25 indicated identity or extensive homology. Database searches were performed with a peptide mass tolerance of 10 ppm, an MS/MS tolerance of 0.5 Da, and a strict tryptic specificity that allowed one missed cleavage site. The carbamidomethylation of cysteine was set as a fixed modification, and oxidation (M) was considered a variable modification.

### Immunocytochemistry

Cells grown on glass coverslips in a six-well plate were treated with 2 μM CAMP-hMT1A for 1 h. After washing with PBS, the cells were stained with MitoTracker Orange (Molecular Probes, Eugene, OR, USA) at 300 nM final concentration for 10 min in complete medium containing 10% FBS. These cells were fixed with 4% ice-cold paraformaldehyde for 10 min and permeabilized with 0.1% Triton X-100. The cells were then blocked with 1% bovine serum albumin (BSA) in Tris-buffered saline for 1 h at room temperature, followed by incubation with rabbit polyclonal 6xHis antibody (1:500; Cell Signaling Technology, Beverly, MA, USA). After washing, the cells were probed with the appropriate secondary antibody conjugated to Alexa Fluor 488 (1:1000; Molecular Probes) for 1 h at room temperature. Nuclei were stained by incubating with Hoechst 33342 (1 μg/ml; Molecular Probes) in PBS for 5 min at room temperature. The slides were then washed twice with PBS and mounted using DAKO fluorescent mounting medium (DAKO Corporation, Carpinteria, CA, USA). The specimens were viewed using a laser-scanning confocal microscope (Carl Zeiss, Oberkochen, Germany) at 405, 488, and 555 nm for Hoechst, 6xHis, and MitoTracker, respectively.

### Isolation of mitochondria

Mitochondria from cultured SK-Hep1 cells were prepared by differential centrifugation. Briefly, the cells were harvested and homogenized in 1 ml of mitochondrial isolation buffer (MIB; 0.25 M sucrose, 0.025 M Tris, and 1 mM EDTA, pH 7.4). The cell homogenate was subjected to centrifugation at 900 × *g* for 10 min, and the supernatant was centrifuged at 8700 × *g* for 10 min. The mitochondria pellet was resuspended in MIB, and the protein concentration was determined by the BCA method (Pierce, Rockford, IL, USA).

### Western blot analysis

Whole-cell (30 μg) or mitochondrial (20 μg) lysates were separated by 15% SDS-PAGE and analyzed by western blot and an enhanced chemiluminescence system (Amersham Bioscience, Piscataway, NJ, USA). Primary antibodies against 6xHis (1:1000, Cell Signaling Technology), MT (1:1000, Santa Cruz, Dallas, TX, USA), Hsp60 (1:1000, Santa Cruz), and tyrosine hydroxylase (TH; 1:1000, Merck Millipore, Billerica, MA, USA) were purchased from commercial sources. Horseradish peroxidase-conjugated secondary antibodies were purchased from Cell Signaling Technology. Equivalent loading of lysate was verified by the anti-β-actin antibody (Sigma Co.). All uncropped scans of the western blots are shown in Supplementary Fig. 1.

### Methylthiazol tetrazolium assay

The 3-(4,5 dimethylthiazol-2-yl)-2,5-diphenyl-tetrazolium bromide (MTT) assay is a measure of mitochondria NADH dehydrogenase complex 1 activity of OXPHOS in live cells. SH-SY5Y cells (1 × 10^5^ cells/well) cultured in 96-well plates in DMEM/F12 containing 0.5% FBS were treated with 1 mM 1-methyl-4-phenyl-2,3-dihydropyridinium ion (MPP^+^) for 24 h. The cells were then incubated with purified CAMP-MT1A for 24 h and further incubated with 0.2 mg/ml MTT (Sigma Co., St. Louis, MO) in PBS for 4 h. The MTT formazan precipitates formed by live cells were dissolved in 100 μl of 0.04 N HCl/isopropanol. The absorbance at 540 nm was measured by a microplate reader (Molecular Devices, Sunnyvale, CA, USA).

### Intracellular ATP measurements

We measured the intracellular ATP concentration via a luciferin-luciferase reaction using CellTiterGlo^®^ Luminescent reagent (Promega), as recommended by the manufacturer. Briefly, 100 μl of cell lysate was mixed with 100 μl of the luciferin-luciferase reaction buffer and incubated at 20 °C for 10 min. We measured the luminescence signal with an LB 9501 Lumat luminometer (Berthold, Badwildbad, Germany). The values were normalized by subtracting the background luminescence value obtained from control wells containing medium without cells. The ATP content was normalized to the protein concentration. All data are presented as the percent of control.

### Oxygen consumption rate

The oxygen consumption rate (OCR) was determined by Oxygraph-2K (Oroboros, Innsbruck, Austria) using mitochondria isolated by differential centrifugation^30^. Approximately 400 µg of mitochondrial protein was suspended in 1 ml of MiRO5 respiration buffer (0.5 mM EGTA, 3 mM magnesium chloride-hexahydrate, 60 mM K-lactobionate, 20 mM taurine, 10 mM potassium phosphate monobasic, 20 mM HEPES, 110 mM sucrose, and 1 g/l fatty acid-free BSA, pH 7.0 with KOH). In some cases, the isolated mitochondria were incubated with CAMP-hMT1A recombinant protein, 1 mM MPP^+^, or 1 μM rotenone for 30 min before OCR measurement. After recording a basal respiration rate, the state 2 OCR (resting complex I-supported respiration) and maximal state 3 mitochondrial respiration rates were measured by the sequential addition of substrates (10 mM glutamate and 4 mM malate) and 1.5 mM ADP, respectively. The OCR was expressed as pmol/s/mg protein.

### Mitochondrial superoxide determination

Mitochondrial superoxide levels were measured after incubating the cells with 5 μM MitoSOX (Molecular Probes) for 10 min at 37 °C, protected from light. The cells were then washed with PBS and counterstained with2 μg/ml Hoechst 33342 for 10 min, and the fluorescence intensities were determined at 510/580 nm. The MitoSOX intensity was normalized to the Hoechst 33432 staining. All data are presented as the percent of control.

### MPP^+^-induced cellular PD model

SH-SY5Y human neuroblastoma cells were cultured in 60-mm or 96-well plates for 24 h, followed by incubation in serum-deficient media (SDM; DMEM/F12 containing 0.5% FBS) for 16 h. The cells in SDM were treated with 1 mM MPP^+^ or dimethyl sulfoxide vehicle for 24 h and further incubated with CAMP-hMT1A for 24 h. The cells were harvested to determine TH expression and mitochondrial activities using analyses previously described, such as the MTT assay, intracellular ATP assay, and MitoSOX superoxide quantification.

### MPTP-induced acute PD mouse model and stereotaxic injection

The animal maintenance and treatments were carried out in accordance with the Principles of Laboratory Animal Care (NIH publication No. 85-23, revised 1996) and the Animal Care and Use guidelines of Kyung Hee University (KHUASP(SE)-16-039), Seoul, Korea. Male C57BL6 mice (8 weeks old, 19–22 g, Daehan Biolink Co., Ltd, Eumseong, Korea) were randomly assigned to three groups (*n* = 7/group): control mice treated with vehicle, MPTP-induced PD model mice treated with vehicle, and MPTP-induced PD model mice treated with CAMP-MT1A (3 μg in PBS). For MPTP intoxication, mice received four intraperitoneal (i.p.) injections of MPTP (20 mg/kg/day, Sigma-Aldrich) dissolved in PBS at 2-h intervals as described^31^. At 9 days after the i.p. injections, these mice were anesthetized by i.p. injection of chloral hydrate (40 mg/kg) and positioned in a stereotaxic apparatus (David Kopf Instruments, Tujunga, CA, USA). A midline sagittal incision was made in the scalp, and holes were drilled in the skull over the SN using the following coordinates: −3.4 mm posterior to bregma and −1.6 mm lateral to the midline for SN injection. The hole of the tip was directed vertically down to 4.6 mm beneath the surface of the brain to reach the SN. All injections (3 μl of PBS vehicle or CAMP-MT1A) were made using a Hamilton syringe equipped with a 30S-gauge needle and attached to a syringe pump (KD scientific, New Hope, PA, USA). Following surgery, the mice were individually housed with food and water available ad libitum for 1 week.

### Rotarod performance test

Mice were evaluated on the rotarod device at 7 days after the MPTP injections to assess sensorimotor coordination. Initially, the mice were required to perch on the stationary rod for 30 s to acclimate them to the environment. The animals were then pre-trained on the rotarod apparatus to reach a stable performance. The training included three separate test trials at 20 rpm rotation speed each lasting 300 s immediately prior to sacrifice. The data are shown as the mean time on the rotating bar over the three test trials.

### Immunohistochemistry

After the rotarod tests, each mouse was transcardially perfused with a saline solution containing 0.5% sodium nitrate and heparin (10 U/ml) and then fixed with 4% paraformaldehyde dissolved in 0.1 M phosphate buffer. The brains were dissected from the skull and post-fixed overnight in buffered 4% paraformaldehyde at 4 °C. The brains were stored in a 30% sucrose solution for 2–3 days at 4 °C until they sank and were frozen sectioned on a sliding microtome in 30-μm-thick coronal sections. All sections were collected in six separate series and processed for immunohistochemical staining as described previously^31^. The dopaminergic neurons were stained with rabbit antibody against TH (1:1000, Merck Millipore), followed by processing with biotinylated secondary antibody and an avidin-biotin complex kit (Vectastain ABC kit; Vector Laboratories, Burlingame, CA, USA). Immunostaining was visualized with 3,3′-diaminobenzidine-HCl, and tissue sections were mounted on gelatin-coated slides and analyzed under a bright-field microscope (Olympus, Tokyo, Japan). The results were quantified by manually counting the number of TH-immunopositive neurons in the SNpc in brain tissue sections at ×100 magnification. TH-immunopositive neurons in the ST were measured by the optical density of TH-positive fibers at ×40 magnification^32^.

### Statistical analysis

The data shown are expressed as the mean ± standard error of the mean. Statistical significance was evaluated by Student’s two-tailed *t*-test, and *P* < 0.05 was considered significant.

## Results

### Effect of cargo proteins on the mitochondrial-targeting probability

The mitochondrial import probability of several virtual constructs consisting of various PTDs conjugated with natural MTSs and cargo proteins was evaluated in silico using the MitoProtII prediction program^26^. The six PTD candidates were *HIV-1* TAT^13^, PTD-4^33^, Pep-1^34^, transportan^35^, *Drosophila* Antennapedia homeodomain Antp^36^, and the herpes virus VP22^37^ (Supplementary Table 1). The MTS candidates used were from three natural human mitochondrial matrix proteins—the N-terminal signal peptide of mMDH, succinate dehydrogenase subunit α (SDHA), and mitochondrial aldehyde dehydrogenase (ALDH2)—in addition to one sMTS used in our previous research^25^ (Supplementary Table 1).

The cationic amino-acid content of the PTDs and MTSs used ranged from 15 to 70%. The predicted probability scores for mitochondrial targeting of virtual fusion proteins with enhanced green fluorescent protein (EGFP) or hMT1A^16,25^ added as a cargo are summarized in Supplementary Table 2. Based on these scores, Pep-1 was a poor PTD for mitochondrial targeting of both EGFP and hMT1A and was therefore excluded from further analysis. When the cargo protein was EGFP, the targeting probability scores of all fusions containing the MTS were over 90%, and the scores were over 57%, even for the constructs lacking MTS. However, when hMT1A was the cargo, all probability scores were <90%, suggesting that an efficient MTS would be necessary for mitochondrial transport of hMT1A (Supplementary Table 2). For the mitochondrial targeting of hMT1A, the best PTD-MTS combination was *HIV-1* TAT connected to the MTS of ALDH2, with a probability score of 87.6% and cationic ion content of 44.8%. When the basic amino-acid content was increased in this virtual construct, the mitochondrial-targeting probability was not improved (data not shown).

### In silico design of CAMP

**Fig. 1:**
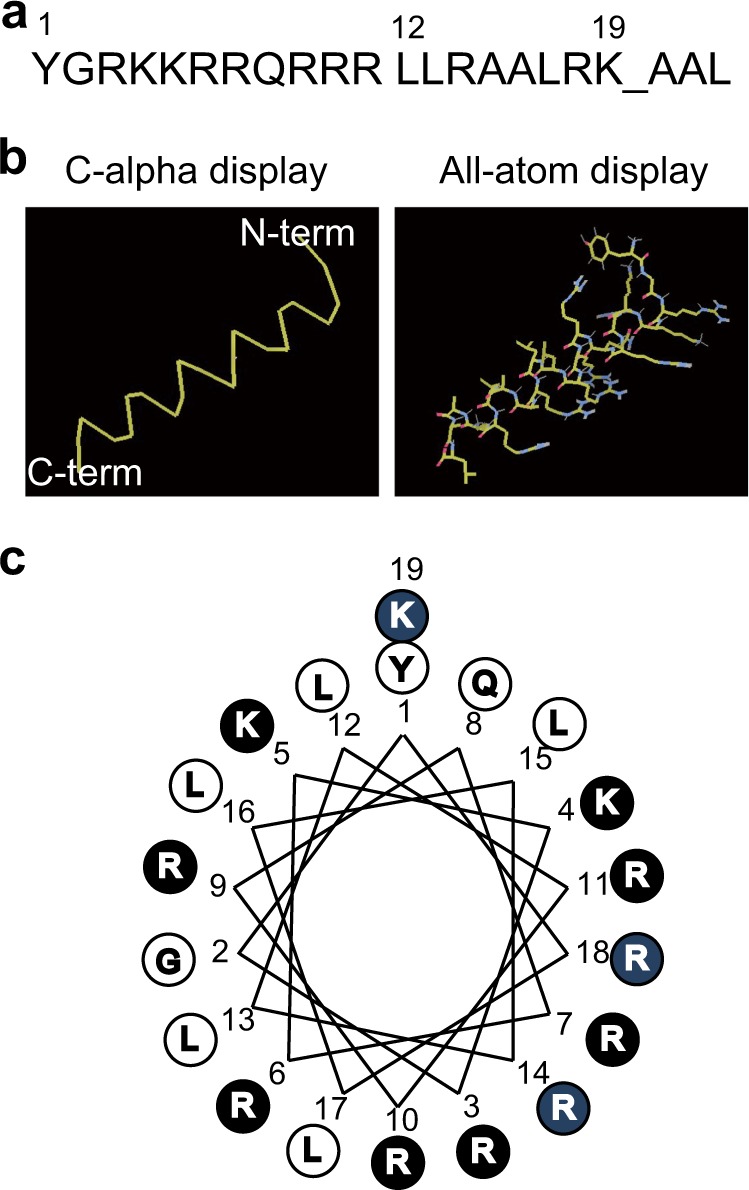
Schematic structure of in silico-designed CAMP peptide. **a** Amino-acid sequence of the CAMP peptide. The cleavage site predicted by MitoProtII is marked with an underscore. **b** α-Helical structure of CAMP predicted by COOT. **c** Helical wheel projection of CAMP. Positively charged amino acids in TAT are presented in black, and those in the MTS are blue

To improve the delivery of therapeutic hMT1A protein into mitochondria, we designed a CAMP in silico. Because most natural MTSs form amphiphilic helices without obvious conserved amino-acid sequences^38,39^, we shuffled and serially truncated the sequences of MTS^ALDH2^ or MTS^SDHA^ in silico and calculated their mitochondrial-targeting scores with TAT (YGRKKRRQRRR) as the PTD and hMT1A as the cargo. As shown in Supplemental Table 3, a short artificial MTS (LLRAALRKAAL) showed the highest score of 97.90%. The fusion of the TAT PTD and this artificial MTS (YGRKKRRQRRRLLRAALRKAAL) was named CAMP. Both the TargetP 1.1^40^ and MitoFates^41^ programs predicted a matrix metalloprotease cleavage site in CAMP between Lys19 and Ala20 (Fig. 1a). Using this cleavage site to remove the MTS from a cargo protein should be beneficial for maintaining the bioactivity of cargo proteins by allowing appropriate folding and mitochondrial retention. The LLRAA and LRKAA sequences of CAMP sequences were also predicted to be TOM20 recognition motifs.

Figure 1b shows the α-helical secondary structure of CAMP predicted by the COOT program^42^. Figure 1c shows the helical wheel projection of CAMP, which exhibits a partial amphiphilicity of positively charged and hydrophobic amino acids. Therefore, CAMP possesses the properties conserved among signal sequences that deliver mitochondrial precursor proteins into mitochondria. The same in silico analysis was conducted to investigate whether CAMP could be applied to various cargoes. Cytosolic antioxidant proteins (SOD1, catalase, EPX, GPX) and PD-associated proteins (PARK2 and LRRK2) were virtually conjugated to CAMP. All mitochondrial-targeting probability scores of these candidate proteins were 97.09–99.96% (data not shown).

### Specific import of CAMP-EGFP and CAMP-hMT1A into mitochondria

**Fig. 2:**
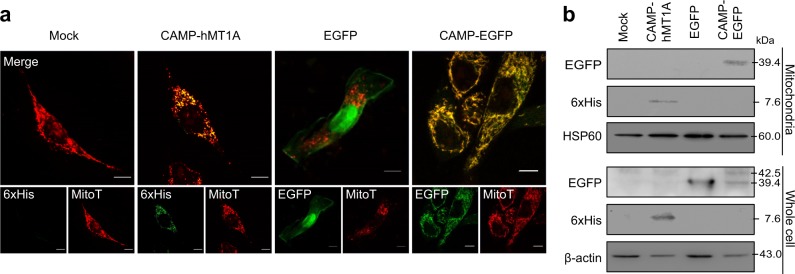
Mitochondrial localization of CAMP-hMT1A and CAMP-EGFP by transfection. SK-Hep1 cells were stably transfected with pcDNA3.1-myc/His (mock), CAMP-hMT1A-6xHis, EGFP, or CAMP-EGFP. **a** Confocal microscopy images showing the localization of CAMP fusion proteins. Cells were stained with MitoTracker (MitoT, red) and immunostained with anti-6xHis or EGFP antibodies (green) before microscopy (×400, scale bar = 10 μm). Co-localization of red and green signal appears yellow in merged images. **b** Western blot analysis. Cell or mitochondrial lysates were analyzed by western blot with 6xHis-tag to detect CAMP-hMT1A and β-actin and HSP60 antibodies for loading and mitochondria controls, respectively

To determine whether CAMP was able to deliver cargo into the mitochondria, mammalian expression plasmids containing hMT1A-6xHis or EGFP with or without CAMP at the N terminus were transfected into SK-Hep1 cells. Immunocytochemistry analysis after MitoTracker staining of the transfected cells demonstrated that hMT1A and EGFP cargo co-localized with the mitochondria (Fig. 2a). Western blot analysis of mitochondrial lysates isolated from the transfected cells revealed that both hMT1A and EGFP were indeed present in the mitochondria (Fig. 2b). Thus, the CAMP peptide was capable of delivering EGFP and hMT1A into mitochondria from the cytoplasm.

### Preparation of CAMP-hMT1A recombinant fusion proteins

**Fig. 3:**
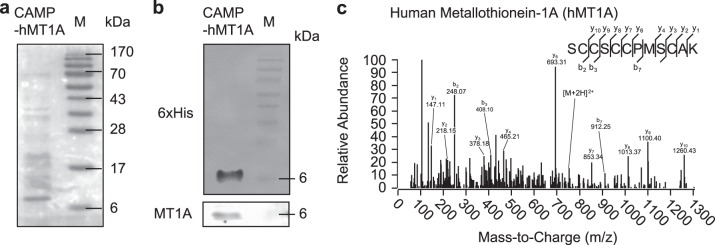
Preparation of CAMP-hMT1A recombinant protein. CAMP-hMT1A protein was expressed in *E. coli* in the presence of 1 mM ZnSO_4_ and purified by Ni-NTA affinity chromatography. **a** Coomassie blue staining. M molecular weight marker. **b** Western blot with 6xHis-tag and metallothionein (MT1A) antibodies to detect CAMP-hMT1A. **c** Mass spectrum analysis. The spectrum of the identified peptide sequence is presented

Next, we prepared recombinant CAMP-hMT1A-6xHis protein, which was expressed in *E. coli* in the presence of 1 mM ZnSO_4_ because zinc has been reported to stabilize MT activity^43^ and were purified by Ni-NTA affinity chromatography. The purity and identity of the CAMP-hMT1A fusion protein were verified by Coomassie blue staining (Fig. 3a) and western blot (Fig. 3b). Mass spectrometry also confirmed that the purified CAMP-hMT1A generated peptides with sequences matching hMT1A (Fig. 3c).

### Mitochondrial targeting of CAMP-hMT1A fusion protein

**Fig. 4:**
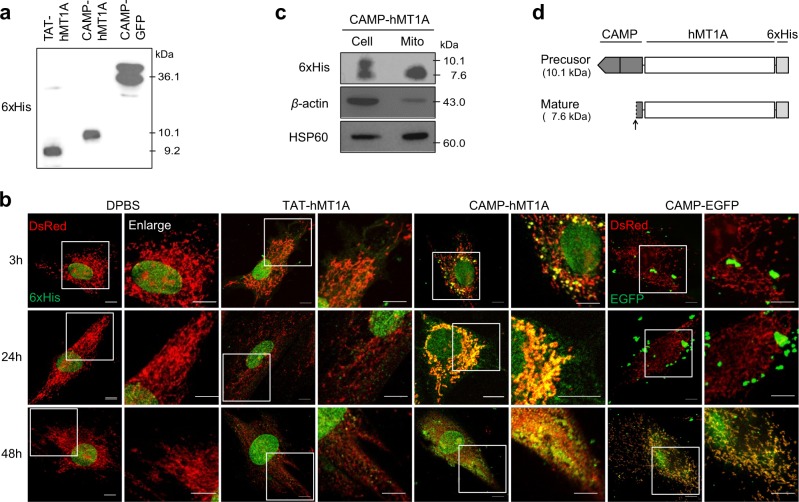
Transfer of CAMP-hMT1A protein into mitochondria. **a** Western blot of TAT-hMT1A, CAMP-hMT1A, and CAMP-EGFP recombinant proteins using 6xHis-tag antibody. **b** Time-dependent uptake of recombinant proteins. SK-Hep1 cells expressing DsRed2-mito (red) were treated with 2 μM CAMP-hMT1A, TAT-hMT1A, or CAMP-EGFP for 3, 24, or 48 h. Cells were immunostained with 6xHis-tag or EGFP antibodies (green) before confocal microscopy (×400, scale bar = 10 μm). The yellow merged images show mitochondrial localization of the fusion proteins. **c** Western blot of CAMP-hMT1A in mitochondria isolated from cells treated with 2 μM CAMP-hMT1A for 24 h. The upper and lower bands of CAMP-hMT1A represent the precursor and mature forms, respectively. β-Actin and HSP60 antibodies were used for loading and mitochondria control, respectively. **d** Schematic representation of the precursor and mature forms of CAMP-hMT1A. The processing site is indicated by an arrow

To investigate the effects of CAMP on mitochondrial transduction efficiency and the intracellular retention time of hMT1A, intracellular uptake and mitochondrial targeting of TAT-hMT1A-6xHis, CAMP-hMT1A-6xHis, and CAMP-EGFP-6xHis recombinant fusion proteins were examined over time by immunocytochemical staining in SK-Hep1 cells expressing DsRed2-mito. The identity of the recombinant protein was confirmed by western blot (Fig. 4a). CAMP-hMT1A was found in mitochondria as early as 3 h after incubation, whereas the TAT-hMT1A construct without the MTS was found only in the cytosol (Fig. 4b). At 24 h, most CAMP-hMT1A protein co-localized with the mitochondria, but TAT-hMT1A remained in the cytosol, and its levels decreased. CAMP-EGFP, which has a high molecular weight, was found in mitochondria after 48 h of incubation.

### CAMP is cleaved from cargo hMT1A protein in mitochondria

When SK-Hep1 cells were incubated with 2 μM recombinant CAMP-hMT1A for 24 h, CAMP-hMT1A was found in isolated mitochondria (Fig. 4c). Western blot analysis revealed that two different CAMP-hMT1A bands (10.1 and 7.6 kDa) were present in whole-cell lysates, but only one protein band (7.6 kDa) was found in mitochondria. If the CAMP peptide were cleaved from hMT1A at the predicted site, the calculated molecular weight of the processed hMT1A should be 7.6 kDa (Fig. 4d). These results suggest that CAMP was properly processed by mitochondrial metalloprotease to produce mature hMT1A.

### CAMP-hMT1A increased mitochondrial function without cytotoxicity

**Fig. 5:**
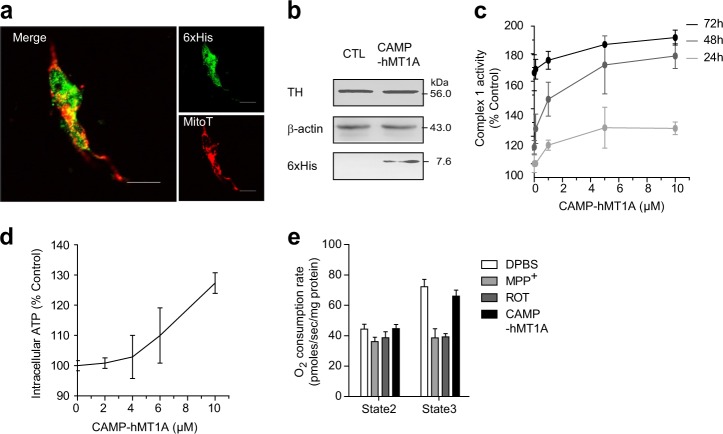
CAMP-hMT1A increases mitochondrial function in neuronal cells without cytotoxicity. **a** Confocal microscopy images of CAMP-hMT1A in the mitochondria of SH-SY5Y neuronal cells (×400, scale bar = 10 μm). Cells were treated with 2 μM CAMP-hMT1A protein for 24 h, stained with MitoTracker (MitoT, red), and immunostained with 6xHis-tag antibody (green). **b** Western blot of tyrosine hydroxylase (TH) levels in CAMP-MT1A- or PBS-treated cells. β-Actin was detected as a loading control. **c** Time- and dose-dependent effects of CAMP-hMT1A on complex 1 of OXPHOS, NADH dehydrogenase activity. **d** Dose-dependent effects of CAMP-hMT1A on intracellular ATP content. **e** The oxygen consumption rate (OCR) of isolated mitochondria for state 2 (–ADP) and state 3 (+1.5 mM ADP) respiration were measured, with mitochondrial complex 1 inhibitors MPP^+^ (1 mM) and rotenone (1 μM) used as positive controls. The data are presented as the mean ± SEM, *n* = 3

The potential toxic effects of CAMP-hMT1A were examined by treating SH-SY5Y neuroblastoma cells with the recombinant CAMP-hMT1A fusion protein. First, 2 μM CAMP-hMT1A was incubated with SH-SY5Y cells for 24 h, and its localization and cytotoxicity were then evaluated. CAMP-hMT1A was translocated into the mitochondria to a similar extent as in SK-Hep1 cells, as observed by immunocytochemical staining (Fig. 5a) and western blot (Fig. 5b). Additionally, the expression level of TH, a rate-limiting enzyme for DA synthesis and a survival marker for dopaminergic neurons, was not altered by CAMP-hMT1A treatment (Fig. 5b). Treatment with CAMP-hMT1A increased mitochondrial NADH dehydrogenase activity in a dose- and time-dependent manner (Fig. 5c) and increased intracellular ATP levels in a dose-dependent manner (Fig. 5d). The OCR of the CAMP-hMT1A-treated cells was not significantly changed in the absence (state 2 respiration) or presence (state 3 respiration) of 1.5 mM ADP (Fig. 5e). These results suggest that CAMP-hMT1A treatment increased mitochondrial function without cytotoxicity.

### Restoration of TH expression and mitochondrial activity by CAMP-hMT1A in PD cell models

**Fig. 6:**
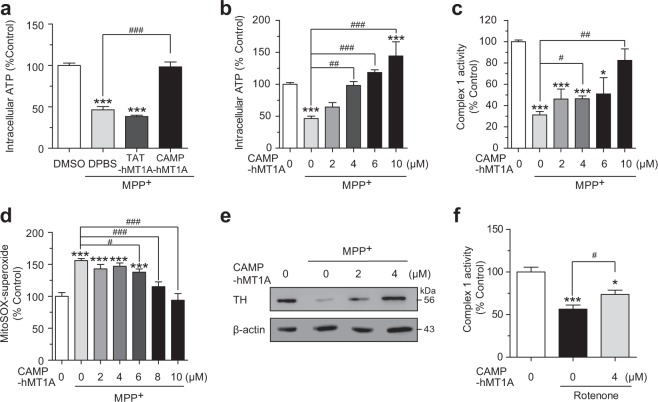
Recovery of mitochondrial activity and TH expression by CAMP-hMT1A in cellular PD models. **a** Intracellular ATP content of SH-SY5Y cells treated with 1 mM MPP^+^ for 24 h, followed by treatment with 4 μM CAMP-hMT1A or TAT-hMT1A (*n* = 4). **b**–**e** Dose-dependent effects of CAMP-hMT1A in MPP^+^-treated SH-SY5Y cells: **b** intracellular ATP (*n* = 12); **c** complex 1 of OXPHOS, NADH dehydrogenase activity (*n* = 4); **d** superoxide level as determined by MitoSOX (*n* = 6); **e** TH expression as determined by western blot with β-actin loading control. **f** Effects of CAMP-hMT1A on complex 1 activity in cells treated with 1 μM rotenone for 24 h (*n* = 3). The data are presented as the mean ± SEM. *P < 0.05, **P < 0.01, ***P < 0.001 vs. CTL; ^#^*P* < 0.05, ^##^*P* < 0.01, ^###^*P* < 0.001 vs. MPP^+^-treated

The therapeutic efficacy of CAMP-hMT1A was examined using MPP^+^-treated SH-SY5Y cells. We treated cells with 1 mM MPP^+^ for 24 h prior to CAMP-hMT1A treatment and monitored recovery from the MPP^+^-mediated damage. CAMP-hMT1A reversed the MPP^+^-mediated decrease of ATP content (*P* < 0.0001, vs. MPP+ treated), whereas TAT-hMT1A did not (Fig. 6a). In fact, CAMP-hMT1A dose-dependently restored ATP (Fig. 6b), mitochondrial NADH dehydrogenase activity (Fig. 6c), and mitochondrial superoxide (Fig. 6d) to levels similar to those of non-MPP+-treated cells. Notably, the TH expression fully recovered upon treatment with 4 μM CAMP-hMT1A (Fig. 6e). Similarly, we tested whether 4 μM CAMP-hMT1A could restore other mitochondrial toxin-induced damage. Figure 6f shows that CAMP-hMT1A also ameliorated the rotenone-induced reduction (*P* < 0.05, vs. rotenone-treated) in mitochondrial NADH dehydrogenase activity. This suggests that CAMP-hMT1A might be a strong candidate to enhance dopaminergic neuron survival by promoting mitochondrial activities.

### Stereotaxic injection of CAMP-hMT1A restored nigrostriatal DA neuron function in MPTP-injected PD mice

**Fig. 7:**
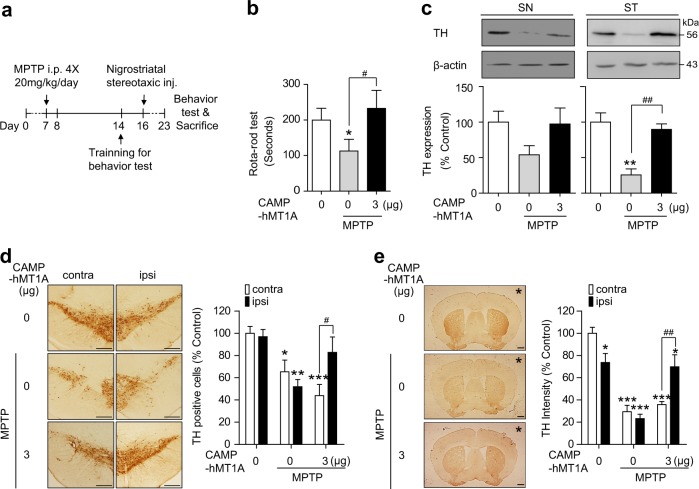
Sensorimotor function and TH expression are restored by stereotaxic injection of CAMP-hMT1A into SN of MPTP-injected mice. **a** Timeline of the MPTP-induced PD model experiment. CAMP-hMT1A (3 μg) or PBS was stereotaxically injected unilaterally into the left substantia nigra. **b** Rotarod performance test (*n* = 7). **c** Western blot and quantification of band intensities of TH in the left substantia nigra (SN) or the striatum (ST) (*n* = 3). β-Actin was the loading control. **d** Immunohistochemistry of TH and relative number of TH-positive cells in the SN (*n* = 4) (×4, scale bar = 1 mm). **e** Immunohistochemistry of TH and relative density of TH-positive fibers in the striatum (×4, scale bar = 1 mm) (*n* = 4, *stereotaxic injection site). The data are presented as the mean ± SEM. ipsi ipsilateral side, contra contralateral side; *P < 0.05, ***P < 0.001 vs. control. ^###^*P* < 0.001 vs. MPTP-treated

The in vivo effects of CAMP-hMT1A were evaluated by stereotaxic injection of 3 μg of CAMP-hMT1A protein into the SN of MPTP-induced acute PD model mice, as shown in Fig. 7a. The rotarod performance test was used to monitor motor deficits, and CAMP-hMT1A injection recovered motor function in MPTP-treated mice (Fig. 7b). Stereotaxically injected CAMP-hMT1A completely recovered TH expression in the SN and ST of MPTP-injected mice, as visualized by western blot (Fig. 7c) and immunostaining (Fig. 7d, e). Collectively, these results indicate that the CAMP-hMT1A fusion protein had a therapeutic effect in both the cellular and animal models of PD.

## Discussion

In the present study, we have developed a peptide containing both a functional cell-penetrating PTD and mitochondria-targeting MTS, which we named CAMP, and demonstrated its ability to deliver therapeutic proteins to treat mitochondrial dysfunction. Conjugation of the hMT1A antioxidant protein to CAMP enabled efficient delivery into mitochondria after penetrating the cell membrane. In cell culture and mouse models of PD, CAMP-hMT1A rescued most physiological features of PD by restoring defective mitochondrial activities and reducing ROS. The CAMP function was demonstrated by both the transfection of a mammalian expression plasmid containing CAMP-hMT1A and the application of recombinant CAMP-hMT1A. The recombinant protein showed excellent transduction efficiency and prolonged intracellular retention time (Fig. 4), probably up to 72 h (Fig. 5). Furthermore, CAMP is proteolytically processed from the cargo protein within the mitochondria, which may be important for the function and half-life of hMT1A because the extra 22 amino acids of CAMP may alter the antioxidant activity of the small hMT1A protein.

PTDs are a class of diverse peptides, 5–30 amino acids in length, with cationic, amphiphilic, and hydrophobic properties that can internalize intact bioactive cargo such as small molecules, plasmid DNA, small interfering RNA, proteins, and nanoparticles across the cell membrane. Considerable attention has been given to PTDs because they have potential use in diagnostic and therapeutic applications due to their high transduction efficiency and low cytotoxicity. Over the past few decades, many preclinical and clinical trials have evaluated various PTD-conjugated peptide therapeutics and found them to be successful in treating a variety of conditions, including cerebral ischemia, Alzheimer’s disease, cancer, and transplant rejection^44,45^. However, there are several drawbacks of using PTDs in clinical settings: (1) their general lack of cell and tissue specificity; (2) the short blood plasma half-life of PTDs administered in vivo; and (3) the short intracellular retention time after endocytosis, which could be attributed to endosomal degradation due to their acidic pH.

To enhance the therapeutic effects of PTD-conjugated bioactive cargo after cell penetration, improvements are needed in organelle targeting and intracellular retention time. We hypothesized that mitochondrial targeting of antioxidant proteins could be an efficient strategy to remove ROS at its site of production. In addition, the internalization into the mitochondria might protect PTD-conjugated bioactive cargo from intracellular degradation processes and, as a result, increase their intracellular retention time and therapeutic effects. We have shown previously that the partial targeting of mouse MT1 into mitochondria, although incomplete or unstable, improved its effects on hypoxia and hyperglycemia^25^. To enhance the mitochondrial-targeting efficiency and retention time, we designed a novel artificial peptide, CAMP, for both cell penetration and mitochondrial targeting.

When we used EGFP as a cargo, most mitochondrial-targeting probability scores of the TAT-MTS-EGFPs exceeded 90%, which was not helpful for deducing the optimal CAMP sequence (Supplementary Table 2). A previous study showed that TAT-EGFP rapidly enters the mitochondrial matrix of PC12 cells, even in the absence of an MTS^16^. However, TAT is not sufficient to deliver cargo proteins other than EGFP to the mitochondria; we previously showed that TAT-conjugated mouse MT1 protein is not efficiently delivered to mitochondria with or without the MTS of mMDH^25^. We used hMT1A as a cargo for calculation because of its future applicability to humans. This allowed us to shuffle and truncate the MTS of ALDH and SDH to predict an effective artificial MTS. The resulting 22-amino-acid CAMP peptide (YGRKKRRQRRRLLRAALRKAAL) shared the characteristics of natural MTS: it formed an amphipathic α-helix rich in basic and hydroxyl residues and lacked acidic residues^46^. Based on our in silico predictions that CAMP will also effectively deliver other cargo to the mitochondria, we conclude that CAMP is theoretically useful for the transduction of many therapeutic cargo proteins across the cell and mitochondrial membranes. This is the first study to develop an artificial peptide with both cell- and mitochondria-penetrating activities in silico using MTS sequences.

Several different pathways have been suggested for the cellular uptake of PTD-cargo conjugates: direct penetration, endocytosis, and micropinocytosis^45^. The cellular uptake mechanism of CAMP-hMT1A was not investigated in the present study. However, the mitochondrial targeting and processing of CAMP-hMT1A suggest that CAMP-hMT1A may enter the cell through the direct penetration pathway rather than through endocytic or macropinocytic pathways, which typically lead to lysosomal degradation. Alternatively, CAMP-hMT1A may escape from the endosomes to the cytosol to avoid degradation and reach the mitochondria. Thus, mitochondrial targeting would be a good strategy to avoid a key limiting factor in the efficient intracellular delivery of functional macromolecules.

Murine MT shows neuroprotective effects as an antioxidant through –SH moieties on cysteine residues and by augmenting glutathione function^47^. Murine MT overexpression inhibits SIN-1 (3-morpholinosydnonimine)-, MPP^+^-, or peroxynitrite-induced oxidative stress and apoptosis to provide neuroprotection^47,48^. We used hMT1A instead of murine MT^25^ to facilitate future applications to humans. Although TAT-hMT1A was less effective than CAMP-hMT1A for recovering mitochondrial activity in our studies, TAT-MT (murine) prevents diabetic neuropathy^49^ and diabetes^50^ through free radical scavenging. Recombinant CAMP-hMT1A reversed damage to mitochondrial ATP production, complex 1 activity, and TH expression, and reduced mitochondrial ROS levels in the MPP^+^/MPTP-induced PD disease model (Figs. 6 and 7). It should be noted that the effects of CAMP-hMT1A manifested in models in which PD had already developed. This finding suggests that CAMP-hMT1A may be effective in late-stage PD patients, unlike other neuroprotective agents. Our results demonstrated that the mitochondrial localization of CAMP-hMT1A might enhance its therapeutic reduction of ROS generation and mitochondrial dysfunction. There are several reports showing that PTD-conjugated therapeutics can cross the blood-brain barrier and penetrate the brain parenchyma, thereby reaching their therapeutic targets and treating cerebral ischemia or Alzheimer’s disease^45^. Thus, the effect of CAMP-hMT1A administered by systemic injection rather than stereotaxic injection should be studied in PD animal models in the near future. In conclusion, CAMP-hMT1A fusion protein delivery into mitochondria might be a promising PD therapy that alleviates mitochondrial damage. In addition to the field of PD treatment, there may be many other potential therapeutic applications of this novel protein transduction technology.

## Electronic supplementary material

Supplemental Materials

Supplemental Tables
