# Supplementary material for: Cell-penetrating artificial mitochondria-targeting peptide-conjugated metallothionein 1A alleviates mitochondrial damage in Parkinson’s disease models
Source: Exp Mol Med. 2018 Aug 17;50(8):105. doi: 10.1038/s12276-018-0124-z (PMC6098059; doi:10.1038/s12276-018-0124-z)
Supplement: Supplementary file 1 — Supplemental Materials [file 12276_2018_124_MOESM1_ESM.pdf]

Supplementary Figure 1a. Full uncropped images

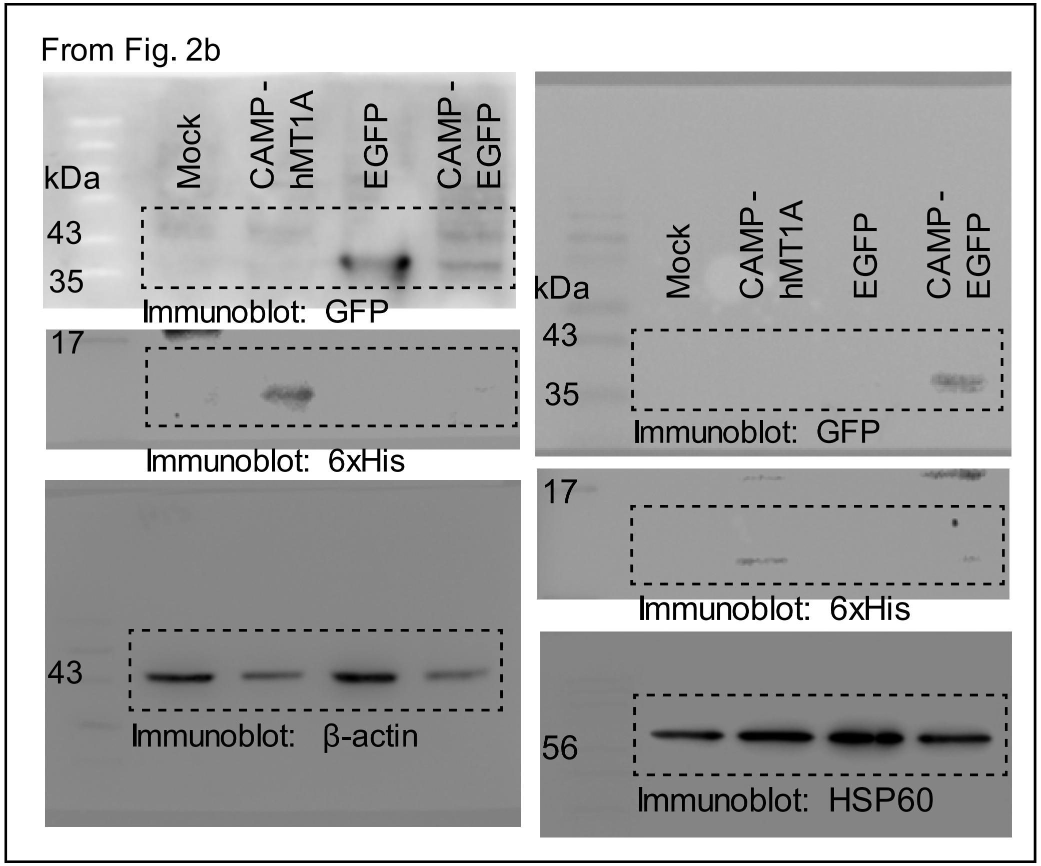

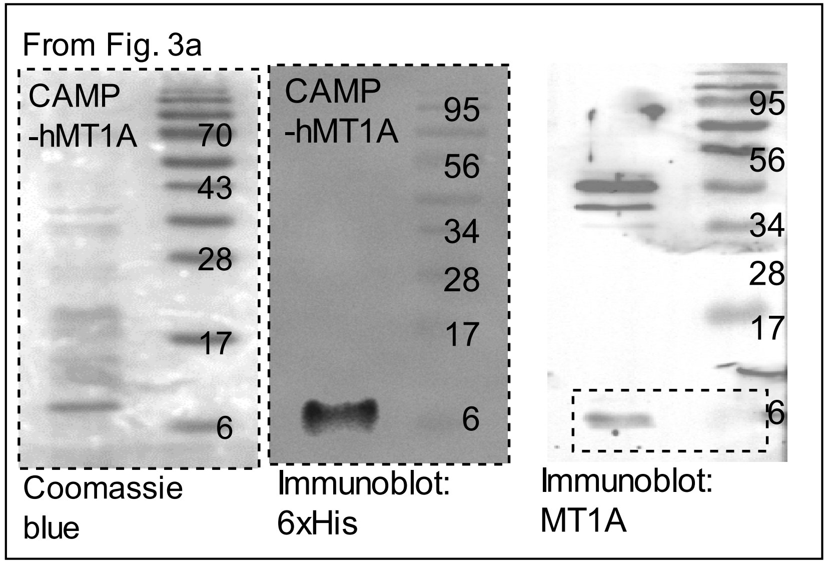

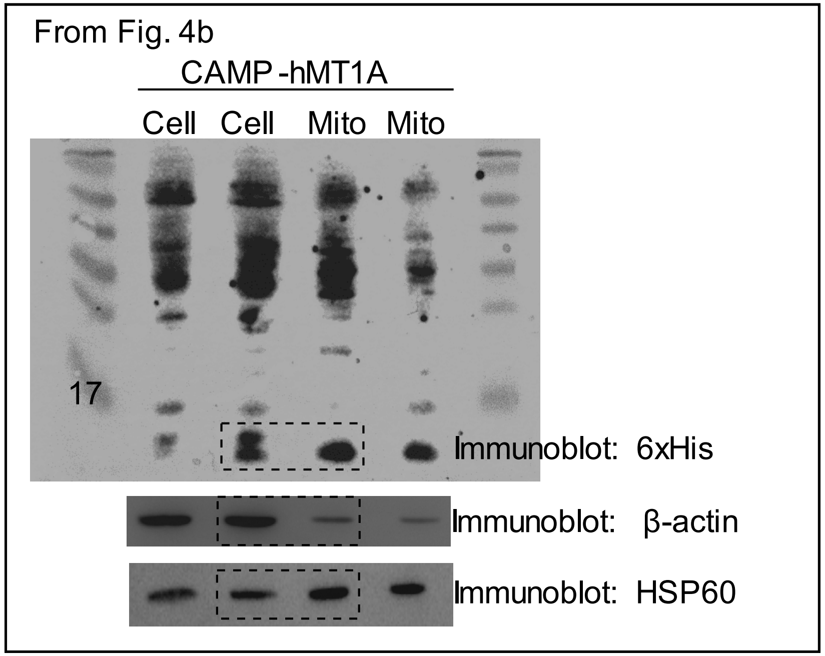

Dashed box represents the cropped images used in figures.

Supplementary Figure 1b. Full uncropped images

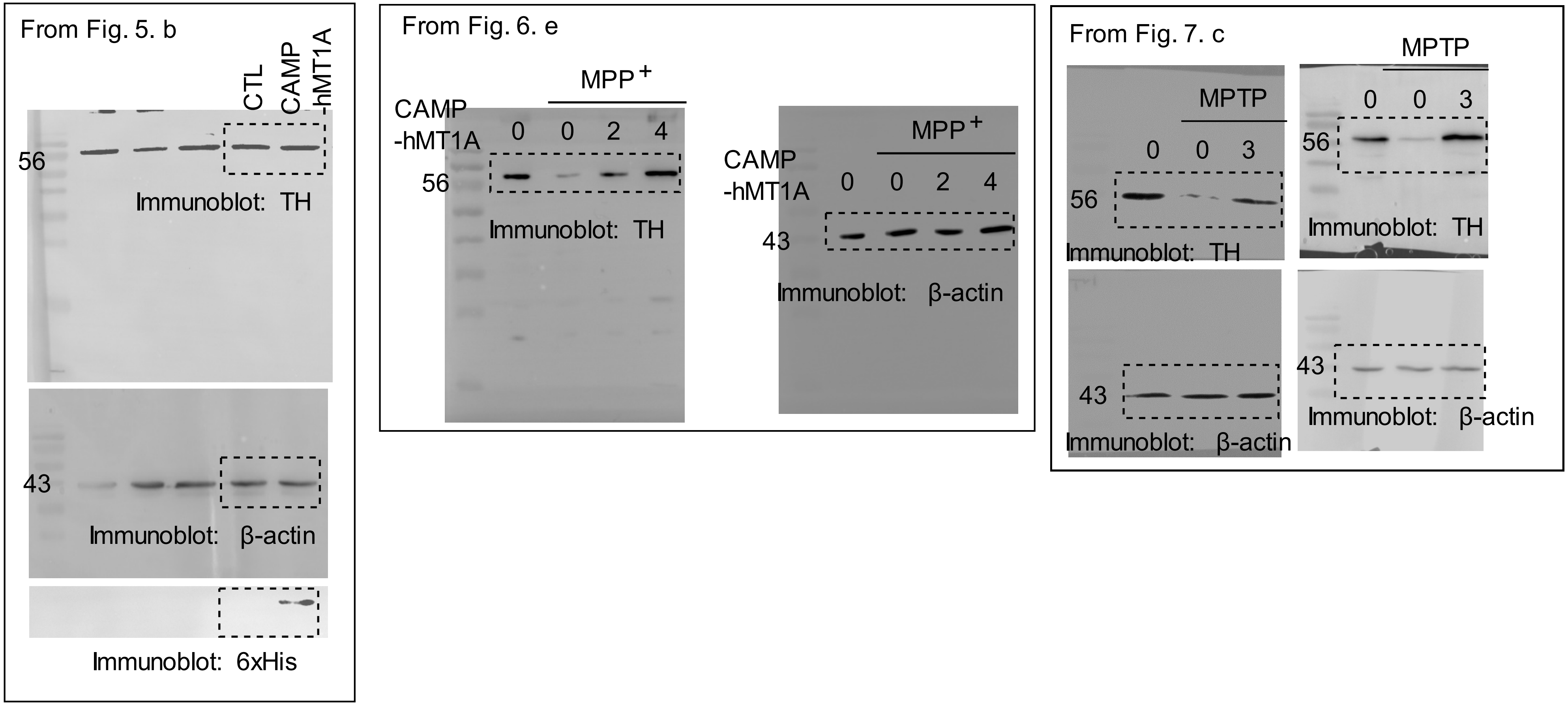

Dashed box represents the cropped images used in figures.
