# Supplementary material for: Cell-penetrating artificial mitochondria-targeting peptide-conjugated metallothionein 1A alleviates mitochondrial damage in Parkinson’s disease models
Source: Exp Mol Med. 2018 Aug 17;50(8):105. doi: 10.1038/s12276-018-0124-z (PMC6098059; doi:10.1038/s12276-018-0124-z)
Supplement: Supplementary file 2 — Supplemental Tables [file 12276_2018_124_MOESM2_ESM.docx]

Supplementary Table 1. Amino acid sequences and cationic amino acid compositions of characterized PTD and MTS

| Name | Sequence | # AA | | Cationic AA | | | |
| --- | --- | --- | --- | --- | --- | --- | --- |
|  |  |  |  | # | | % | |
| **PTD** |  | |  | |  | |  |
| TAT | YGRKKRRQRRR | | 11 | | 8 | | 72.7 |
| PTD-4 | YARAAARQARA | | 11 | | 3 | | 27.3 |
| Pep-1 | KETWWETWWTEWSQPKKKRKV | | 21 | | 6 | | 28.6 |
| Transportan | GWTLNSAGYLLGKINLKALAALAKKIL | | 27 | | 4 | | 14.8 |
| Antp | RQIKIWFQNRRMKWKK | | 16 | | 7 | | 43.8 |
| VP22 | DAATATRGRSAASRPTERPRAPARSASRPRRPVE | | 34 | | 8 | | 23.5 |
| **MTS** |  | |  | |  | |  |
| mMDH | MLSALARPVGAALRRSFSTSAQNN | | 24 | | 3 | | 12.5 |
| sMTS | MVSAL | | 5 | | 0 | | 0 |
| SDHA | MSVLTPLLLRGLTGSARRLPVPRAKIHSL | | 28 | | 6 | | 21.4 |
| ALDH2 | MLRAALSTARKGPRLSRL | | 18 | | 5 | | 27.8 |

Abbreviations: PTD, protein transduction domain; MTS, mitochondria-targeting sequence; TAT, trans-activator of transcription; Antp, *Drosophila* Antennapedia homeodomain; VP22, the herpesvirus protein VP22; mMDH, mitochondria malate dehydrogenase; sMTS, a five amino acid-short peptide derived from MTS of mMDH; SDHA, succinate dehydrogenase subunit α; ALDH2, mitochondrial aldehyde dehydrogenase; AA, amino acid

Supplementary Table 2. The probability scores (%) for mitochondrial targeting of the virtual PTD-MTS peptides-conjugated to cargo proteins. The scores were calculated *in silico* using MitoProtII prediction program.

| PTD | MTS | Cargo proteins | | | PTD-MTS | | |
| --- | --- | --- | --- | --- | --- | --- | --- |
|  |  | None | EGFP | hMT1A | #AA | #Cation | %Cation |
| TAT | None | 0 | 61.7 | 3.19 | 11 | 8 | 0 |
|  | mMDH | 96.59 | 99.89 | 74.27* | 35 | 11 | 31.4 |
|  | sMTS | 0 | 96.31 | 12.07 | 16 | 8 | 50.0 |
|  | SDHA | 98.3 | 99.94 | 77.15 | 39 | 14 | 35.9 |
|  | ALDH2 | 98.54 | 99.98 | 87.6* | 29 | 13 | 44.8 |
| PTD-4 | None | 0 | 77.17 | 2.3 | 11 | 3 | 0 |
|  | mMDH | 90.34 | 99.98 | 61.35 | 35 | 6 | 17.1 |
|  | sMTS | - | 95.9.8 | 13.67 | 16 | 3 | 18.8 |
|  | SDHA | 96.57 | 99.95 | 60.17 | 39 | 9 | 23.1 |
|  | ALDH2 | 88.23 | 99.9 | 59.07 | 29 | 8 | 27.6 |
| Pep-1 | None | 0 | 10.39 | 0.38 | 21 | 6 | 0 |
|  | mMDH | 0.3 | 11.62 | 0.48 | 45 | 9 | 20.0 |
|  | sMTS | 6.09 | 12.95 | 2.48 | 26 | 6 | 23.1 |
|  | SDHA | 0.6 | 13.25 | 1.13 | 49 | 12 | 24.5 |
|  | ALDH2 | 0.74 | 11.65 | 0.39 | 39 | 11 | 28.2 |
| Transportan | None | 0 | 68.46 | 2.41 | 27 | 4 | 0 |
|  | mMDH | 52.26 | 99.91 | 22.9 | 51 | 7 | 13.7 |
|  | sMTS | 0.47 | 49.28 | 1.1 | 32 | 4 | 12.5 |
|  | SDHA | 17.16 | 99.82 | 17 | 55 | 10 | 18.2 |
|  | ALDH2 | 40.02 | 99.91 | 26.9 | 45 | 9 | 20.0 |
| Antp | None | 0 | 57.07 | 1.0 | 16 | 7 | 0 |
|  | mMDH | 96.64 | 99.95 | 43.4 | 40 | 10 | 25.0 |
|  | sMTS | 21.35 | 81.62 | 2.5 | 21 | 7 | 33.3 |
|  | SDHA | 97.23 | 99.99 | 66.6 | 44 | 13 | 29.5 |
|  | ALDH2 | 95.52 | 99.99 | 51.2 | 34 | 12 | 35.3 |
| VP22 | None | 0 | 91.49 | 19.2 | 34 | 8 | - |
|  | mMDH | 77 | 99.41 | 6.1 | 58 | 11 | 19.0 |
|  | sMTS | 21.06 | 92.76 | 35.0 | 39 | 8 | 20.5 |
|  | SDHA | 87.74 | 99.16 | 11.8 | 62 | 14 | 22.6 |
|  | ALDH2 | 46.23 | 99.40 | 1.3 | 52 | 13 | 25.0 |

Supplementary Table 3. Mitochondrial targeting probability scores of the virtual TAT-MTS-hMT1A fusion proteins by MitoProtII program to deduce cell-penetrating artificial MTS (CAMP).

| Sequence of MTS | #AA of MTS | Probability score (%) |
| --- | --- | --- |
| MLRAALSTARK_GPRLSR | 17 | 87.14 |
| MLRAALSTARK_GPRLS | 16 | 58.28 |
| MLRAALSTARK_GPRL | 15 | 71.35 |
| MLRAALSTARK_GPR | 14 | 58.62 |
| MLRAALSTARKGP | 13 | 40.67 |
| MLRAALSTARKG | 12 | 26.90 |
| MLRAALSTARK | 11 | 40.81 |
| MLRAALSTAR | 10 | 26.96 |
| MLRAALSTA | 9 | 63.32 |
| MLRAALST | 8 | 21.77 |
| MLRAALS | 7 | 16.41 |
| -LRAALSTARK_GPRLSRL | 17 | 24.53 |
| --RAALSTARK_GPRLSRL | 16 | 40.39 |
| ---AALSTARK_GPRLSRL | 15 | 43.54 |
| ----ALSTARK_GPRLSRL | 14 | 43.50 |
| -----LSTARK_GPRLSRL | 13 | 40.67 |
| ------STARK_GPRLSRL | 12 | 4.78 |
| -------TARK_GPRLSRL | 11 | 4.90 |
| --------ARK_GPRLSRL | 10 | 47.98 |
| ---------RK_GPRLSRL | 9 | 14.75 |
| ----------K_GPRLSRL | 8 | 0.88 |
| -----------_GPRLSRL | 7 | 6.74 |
| MLRAALSTARK_GPRLSRL | 18 | 87.60 |
| -LRAALSTARK_GPRLSRL | 17 | 24.53 |
| M-RAALSTARK_GPRLSRL | 17 | 22.32 |
| ML-AALSTARK_GPRLSRL | 17 | 29.96 |
| MLR-ALSTARK_GPRLSRL | 17 | 79.87 |
| MLRAA-STARK_GPRLSRL | 17 | 75.07 |
| MLRAAL-TARK_GPRLSRL | 17 | 76.63 |
| MLRAALS-ARK_GPRLSRL | 17 | 76.60 |
| MLRAALST-RK_GPRLSRL | 17 | 75.13 |

| MLRAALSTA-KGPRLSRL | 17 | 72.27 |
| --- | --- | --- |
| MLRAALSTAR-GPRLSRL | 17 | 72.78 |
| MLRAALSTARK-PRLSRL | 17 | 68.78 |
| MLRAALSTARKG-RLSRL | 17 | 76.32 |
| MLRAALSTARKGP-LSRL | 17 | 75.31 |
| MLRAALSTARK_GPR-SRL | 17 | 80.64 |
| MLRAALSTARK_GPRL-RL | 17 | 79.29 |
| MLRAALSTARK_GPRLS-L | 17 | 60.51 |
| MLRAALSTARK_GPRLSR- | 17 | 87.14 |
| --RAALSTARK_GPRLSRL | 16 | 40.39 |
| M_--AALSTARKGPRLSRL | 16 | 19.10 |
| ML--ALSTARK_GPRLSRL | 16 | 37.91 |
| MLR--LSTARK_GPRLSRL | 16 | 53.44 |
| MLRA--STARK_GPRLSRL | 16 | 60.02 |
| MLRAA--TARK_GPRLSRL | 16 | 58.25 |
| MLRAAL--ARK_GPRLSRL | 16 | 90.69 |
| MLRAALS--RK_GPRLSRL | 16 | 93.24 |
| MLRAALST--KGPRLSRL | 16 | 59.76 |
| MLRAALSTA--GPRLSRL | 16 | 87.00 |
| MLRAALSTAR--PRLSRL | 16 | 88.48 |
| MLRAALSTARK--RLSRL | 16 | 91.92 |
| MLRAALSTARKG--LSRL | 16 | 94.93 |
| MLRAALSTARK_GP--SRL | 16 | 74.32 |
| MLRAALSTARK_GPR--RL | 16 | 79.14 |
| MLRAALSTARK_GPRL--L | 16 | 63.82 |
| MLRAALSTARK_GPRLS-- | 16 | 58.28 |
| ---AALSTARK_GPRLSRL | 15 | 43.54 |
| M---ALSTARKGPRLSRL | 15 | 44.17 |
| ML---LSTARK_GPRLSRL | 15 | 49.64 |
| MLR---STARK_GPRLSRL | 15 | 93.52 |
| MLRA---TARK_GPRLSRL | 15 | 90.38 |
| MLRAA---ARK_GPRLSRL | 15 | 91.14 |
| MLRAAL---RK_GPRLSRL | 15 | 93.59 |
| MLRAALS---KGPRLSRL | 15 | 63.21 |
| MLRAALST---_GPRLSRL | 15 | 45.35 |
| MLRAALSTA---PRLSRL | 15 | 51.74 |
| MLRAALSTAR---RLSRL (MDPN_) | 15 | 85.83 |
| MLRAALSTARK---LSRL (MDPN_) | 15 | 84.85 |
| MLRAALSTARK_G---SRL | 15 | 73.47 |
| MLRAALSTARK_GP---RL | 15 | 71.35 |
| MLRAALSTARK_GPRLSRL | 18 | 87.60 |
| ----ALSTARK_GPRLSRL | 14 | 43.50 |
| M----LSTARKGPRLSRL | 14 | 45.97 |
| ML----STARK_GPRLSRL | 14 | 49.57 |
| MLR----TARK_GPRLSRL | 14 | 83.41 |
| MLRAA----RK_GPRLSRL | 14 | 86.25 |
| MLRAAL----KGPRLSRL | 14 | 64.99 |
| MLRAALS----GPRLSRL | 14 | 61.60 |
| MLRAALST----PRLSRL | 14 | 77.40 |
| MLRAALSTA----RLSRL | 14 | 79.49 |
| MLRAALSTAR----LSRL | 14 | 79.49 |
| MLRAALSTARK----SRL | 14 | 76.59 |
| MLRAALSTARKG----RL | 14 | 76.05 |
| MLRAALSTARK_GP----L | 14 | 40.83 |
| MLRAALSTARKGPR---- (M_) | 14 | 58.62 |
| -----LSTARKGP_RLSRL | 13 | 4.78 |
| M-----STARK_GPRLSRL | 13 | 4.74 |
| ML-----TARKGPR_LSRL | 13 | 5.24 |
| MLR-----ARK_GPRLSRL | 13 | 5.66 |
| MLRA-----RK_GPRLSRL | 13 | 5.66 |
| MLRAA-----KGPRLSRL | 13 | 5.83 |
| MLRAAL-----GPRLSRL | 13 | 66.00 |
| MLRAALS-----PRLSRL | 13 | 74.58 |
| MLRAALST-----RLSRL | 13 | 79.96 |
| MLRAALSTA-----LSRL | 13 | 75.67 |
| MLRAALSTAR-----SRL | 13 | 67.38 |
| MLRAALSTARK-----RL | 13 | 81.92 |
| MLRAALSTARKG-----L | 13 | 46.04 |
| MLRAALSTARKGP----- | 13 | 46.07 |
| ------STARK_GPRLSRL | 12 | 4.90 |
| M------TARK_GPRLSRL | 12 | 6.71 |
| ML------ARKGPRL_SRL | 12 | 21.76 |
| MLR------RK_GPRLSRL | 12 | 63.16 |
| MLRA_------KGPRLSRL | 12 | 27.22 |
| MLRA_A------GPRLSRL | 12 | 59.11 |
| MLRAAL------PRLSRL | 12 | 78.43 |
| MLRAALS------RLSRL | 12 | 83.87 |
| MLRAALST------LSRL | 12 | 57.28 |
| MLRAALSTA------SRL | 12 | 52.42 |
| MLRAALSTAR------RL | 12 | 63.53 |
| MLRAALSTARK------L | 12 | 38.58 |
| MLRAALSTARKG------ | 12 | 26.90 |
| -------TARK_GPRLSRL | 11 | 47.98 |
| M-------ARK_GPRLSRL | 11 | 62.39 |
| ML-------RK_GPRLSRL | 11 | 72.97 |
| MLR-------K_GPRLSRL | 11 | 72.97 |
| MLRA_-------GPRLSRL | 11 | 67.32 |
| MLRA_A-------PRLSRL | 11 | 75.27 |
| MLRAAL-------RLSRL | 11 | 69.84 |
| MLRAALS-------LSRL | 11 | 50.39 |
| MLRAALST-------SRL | 11 | 45.49 |
| MLRAALSTA-------RL | 11 | 50.43 |
| MLRAALSTAR-------L | 11 | 50.43 |
| MLRAALSTARK------- | 11 | 40.81 |
| --------ARK_GPRLSRL | 10 | 14.75 |
| M--------RKGPRLSRL | 10 | 15.00 |
| ML--------KGPRLSRL | 10 | 10.77 |
| MLR--------GPRLSRL | 10 | 17.93 |
| MLRA--------PRLSRL | 10 | 21.88 |
| MLRAA--------RLSRL | 10 | 44.86 |
| MLRAAL--------LSRL | 10 | 78.82 |
| MLRAALS--------SRL | 10 | 73.48 |
| MLRAALST--------RL | 10 | 78.16 |
| MLRAALSTA--------L | 10 | 77.98 |
| MLRAALSTAR-------- | 10 | 26.96 |
| ---------RK_GPRLSRL | 9 | 0.88 |
| M_---------KGPRLSRL | 9 | 2.99 |
| ML---------GPRLSRL | 9 | 12.06 |
| MLR---------PRLSRL | 9 | 54.07 |
| MLRA---------RLSRL | 9 | 19.27 |
| MLRAA---------LSRL | 9 | 83.61 |
| MLRAAL---------SRL | 9 | 83.61 |
| MLRAALS---------RL | 9 | 83.61 |
| MLRAALST---------L | 9 | 62.44 |
| MLRAALSTA--------- | 9 | 63.32 |
| ----------K_GPRLSRL | 8 | 3.97 |
| M----------GPRLSRL | 8 | 7.11 |
| ML----------PRLSRL | 8 | 15.28 |
| MLR----------RLSRL (MDPN_) | 8 | 44.53 |
| MLRA----------LSRL (MDPN_) | 8 | 55.98 |
| MLRAA----------SRL | 8 | 42.65 |
| MLRAAL----------RL | 8 | 31.83 |
| MLRAALS----------L | 8 | 44.85 |
| MLRAALST---------- | 8 | 21.77 |
| LLRAAL---RK_GPRLSRL | 15 | 94.24 |
| ALRAAL---RK_GPRLSRL | 15 | 92.40 |
| QLRAAL---RK_GPRLSRL | 15 | 74.86 |
| GLRAAL---RK_GPRLSRL | 15 | 89.42 |
| YLRAAL---RK_GPRLSRL | 15 | 89.65 |
| -LRAAL---RK_GPRLSRL | 14 | 73.64 |
| MLRAAL---RKGPRLSR- (M_) | 14 | 91.99 |
| LLRAAL---RKGPRLSR- (M_) | 14 | 92.49 |
| ALRAAL---RKGPRLSR- (M_) | 14 | 90.29 |
| LLRAAL---RK_GPRLS-- | 13 | 89.63 |
| ALRAAL---RK_GPRLS-- | 13 | 81.70 |
| -LRAAL---RK_GPRLS-- | 12 | 60.50 |
| LLRAAL---RK_GPRL--- | 12 | 87.96 |
| ALRAAL---RK_GPRL--- | 12 | 84.34 |
| -LRAAL---RK_GPRL--- | 11 | 60.69 |
| LLRAAL---RK_GPR---- | 11 | 88.32 |
| ALRAAL---RK_GPR---- | 11 | 84.68 |
| LLRAAL---RK_GP----- | 10 | 86.80 |
| ALRAAL---RK_GP----- | 10 | 82.99 |
| LLRAAL---RKG------ | 9 | 90.85 |
| ALRAAL---RKG------ | 9 | 89.83 |
| LLRAAL---RK_AA----- | 10 | 93.10 |
| ALRAAL---RK_AA----- | 10 | 91.95 |
| LLRAAL---RKAAR----(M_) | 11 | 91.88 |
| LLRAAL---RK_AA-L--- | 11 | 97.90 |
| LLRAAL---RK_AA-LS-- | 12 | 95.48 |
| LLRAAL---RKAA-LSR- (M_) | 13 | 97.48 |
| LLRAAL---RK_AA-LS-L | 13 | 97.75 |
| LLRLLL---RK_AA----- | 10 | 93.80 |
| ALRLLL---RK_AA---- | 10 | 91.95 |
| LLRLLL---RK_AAA---- | 11 | 95.47 |
| ALRLLL---RK_AAA---- | 11 | 90.83 |
| LLRLLL---RK_AAL---- | 11 | 97.20 |
| ----ALSTARK------- | 7 | 17.44 |
| --PAALSTARK_GPRLSRL | 16 | 23.27 |
| --PAALSTARK------- | 8 | 6.69 |
| -----------GPRLSRL | 7 | 6.74 |
| ---------PRKGPRLSL | 9 | 14.42 |
| --PAALSTARKGPRLSR- (M_) | 15 | 14.10 |
| --PAALSTARKGPRLS-- | 14 | 47.06 |
| --PAALSTATKGPRLSL- | 15 | 29.48 |
| LLRAA---RRK_AAL---- | 11 | 56.19 |
| SVLTPLLLRGLTGSARRLPVPRAKI_HSL | 39 | 77.15 |
| SVLTPLLLRGLTGSARRL----------- | 29 | 88.42 |
| SVLTPLLLRGLTGSA-------------- | 26 | 41.40 |
| ----PLLLRGLTGSARRL----------- | 25 | 51.41 |
| --------RGLTGSARRLPVPR------- | 25 | 24.77 |
| ---------------RRLPVPR------- | 18 | 21.84 |
| ----------LTGSARR------------ | 18 | 27.21 |

NOTE: The probability scores over 90% were highlighted. The MTS sequence (red letter) with the highest score was selected for CAMP peptide. Under bar (_) denotes the hypothetical metalloprotease processing site predicted by TargetP1.1 and MitoFates.
